# Response by Deng et al. Regarding Article, “C‐Reactive Protein–Albumin–Lymphocyte (CALLY) Index as an Independent Risk Factor for Postoperative Atrial Fibrillation Recurrence”

**DOI:** 10.1002/clc.70184

**Published:** 2025-07-21

**Authors:** Ye Deng, Yuan Ji, Ling Sun

**Affiliations:** ^1^ Department of Cardiology The Third Affiliated Hospital of Nanjing Medical University Changzhou Jiangsu China; ^2^ Department of Cardiology The Affiliated Wuxi People's Hospital of Nanjing Medical University Wuxi Jiangsu China

## Conflicts of Interest

The authors declare no conflicts of interest.

Dear Editor,

1

We thank the authors for their thoughtful comments on our article “C‐Reactive Protein–Albumin–Lymphocyte (CALLY) Index as an Independent Risk Factor for Postoperative Atrial Fibrillation Recurrence” [1] and appreciate the opportunity to address their considerations.

First, Repeat ablations were performed in a subset of patients with recurrent AF, during which the status of pulmonary vein isolation was assessed. Regrettably, the sample size of these cases was insufficient to warrant a formal subgroup analysis. However, we hypothesize that a higher CALLY index may indicate inherently poorer atrial substrate properties—specifically, that an activated inflammatory state could facilitate myocardial fibrosis and the formation of reentrant circuits, thereby predisposing to AF recurrence. This hypothesis, of course, necessitates validation through further clinical and preclinical investigations.

Second, we concur that the CALLY index, which incorporates albumin levels, may reflect an activated inflammatory state that encompasses hepatic processes. Accumulating evidence suggests potential crosstalk between the liver and heart in the context of cardiovascular disease: for instance, cardiovascular conditions have been shown to exacerbate liver fibrosis in patients with fatty liver disease, with Ly6Chi monocytes playing a pivotal role [2]. It is reasonable to hypothesize that such liver‐heart interactions may contribute to AF recurrence, potentially mediated by the inflammatory pathways captured by the CALLY index. As you suggested, advanced imaging modalities (e.g., cardiac magnetic resonance imaging) would undoubtedly help elucidate the intricate relationships between the CALLY index, myocardial inflammation, and AF recurrence. This constitutes a pivotal direction for our subsequent research endeavors.

Third, our baseline data revealed no significant association between alcohol consumption and AF recurrence. This is also consistent with previous study [3]. We acknowledge that larger‐scale and multicenter studies are warranted to more thoroughly explore the potential role of alcohol and other lifestyle factors in AF recurrence.

Finally, we thank the authors for their insightful comments, which have improved our work's clarity and interpretative depth, thereby enhancing its clinical value in guiding AF treatment.

## Data Availability

Data sharing not applicable to this article as no data sets were generated or analyzed during the current study.
